# Coronary artery spasm treated with intracoronary bioresorbable scaffold implantation under the guidance of treadmill test and optical coherence tomography: A case report

**DOI:** 10.1111/anec.13037

**Published:** 2023-01-10

**Authors:** Yang Yang, Xinghua Jiang, Jun Guo, Kai Zou, Qianwei Huang, Biming Zhan

**Affiliations:** ^1^ Department of Cardiology The Second Affiliated Hospital of Nanchang University Nanchang China

**Keywords:** bioresorbable scaffold, coronary angiography, coronary artery spasm, optical coherence tomography, therapy

## Abstract

Coronary artery spasm (CAS) can cause unstable angina, and the treatment of this disease is controversial. We report an elderly male patient who was admitted to hospital due to chest tightness. CAG showed that 70% stenosis in the middle of the right coronary artery (RCA). A bioresorbable scaffold (BRS) was implanted in the lesion under the guidance of optical coherence tomography (OCT). One year later, the patient's symptoms were relieved. The repeated CAG showed that the stent was good. BRS implantation under the guidance of treadmill test and OCT is one of treatment options for CAS patients.

## Graphical abstract

A patient was finally diagnosed with coronary artery spasm. The patient was treated by implanting intracoronary bioresorbable scaffold implantation under the guidance of treadmill test and optical coherence tomography.

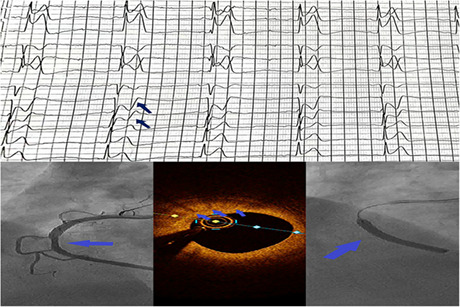

Coronary artery spasm (CAS) was first described in 1959 as “a variant of angina pectoris” (Prinzmetal et al., 1959). The electrocardiogram (ECG) often provides the diagnosis for CAS, which is characterized by ST‐segment elevation consistent with recurrent episodes of angina at rest. CAS can cause myocardial ischemia, acute myocardial infarction, and even lead to malignant arrhythmias, such as atrioventricular block, ventricular fibrillation, and cardiac arrest, leading to sudden cardiac death (Eschalier et al., 2014; Zhang et al., 2015). The optimal treatment of CAS is controversial, and current treatments include calcium channel blockers or nitrates, coronary artery stent implantation, and implantable cardioverter defibrillator implantation (Gaspardone et al., 1999; JCS Joint Working Group, 2014; Lacroix et al., 1994). This is the first study to share the initial experience of intracoronary bioresorbable scaffold (BRS) under the guidance of treadmill test and optical coherence tomography (OCT) in the treatment of CAS patients.

## 1 CASE PRESENTATION

**FIGURE 1:**
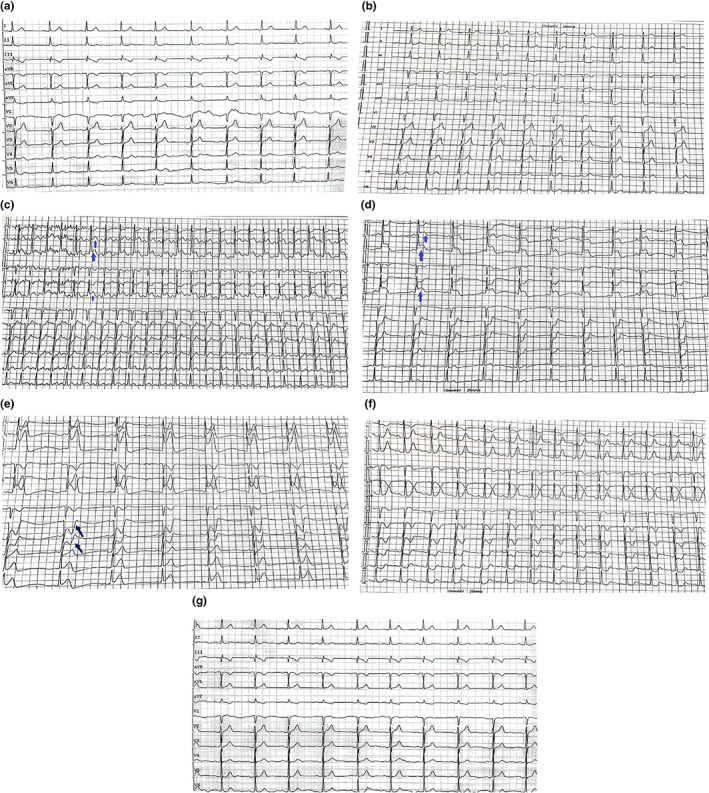
ECG. (a) Standard 12‐lead surface ECG of the patient upon admission. Sinus bradycardia, T wave changes. (b) The ECG of the beginning of the treadmill test. No obvious abnormality was found in each lead. (c) Start exercising under load. ST‐segment elevation in II, III, and avF leads (arrow). (d) Almost reaching maximum load exercise. ST‐segment elevation was more obvious in II, III, and avF leads (arrow). (e) Gradually reducing the load and exercise. ST‐segment continued to change (arrow). (f) The ECG of the treadmill test that is about to stop. ST segment changes also gradually recovered. (g) Standard 12‐lead surface ECG after operation. Sinus heart rate, and roughly normal ECG.

A 62‐year‐old male patient was admitted to the emergency department of the hospital because of recurrent chest tightness for more than 4 months, and then aggravated for 3 h. He had a history of hypertension for 1 year, and took extended release nifedipine tablets, sacubitril‐valsartan sodium tablets and metoprolol succinate sustained‐release tablets to control blood pressure. He smoked for 30 years, 20 cigarettes per day. The patient's blood pressure 124/74 mmHg and heart rate 76 bpm and his physical examination was unremarkable. Initial laboratory tests including blood routine test, blood lipid and muscle enzyme spectrum, liver function, kidney function, serum troponin, electrolytes were generally normal. The ECG showed sinus bradycardia, T wave changes (Figure 1a). Transthoracic echocardiography showed ventricular septal thickening, left ventricular diastolic dysfunction, tricuspid regurgitation, and left ventricular ejection fraction was 60%. He experienced chest tightness, chest pain, and the ECG showed ST‐segment elevation in II, III, and avF leads during the treadmill test (Figure 1b–f).

**FIGURE 2:**
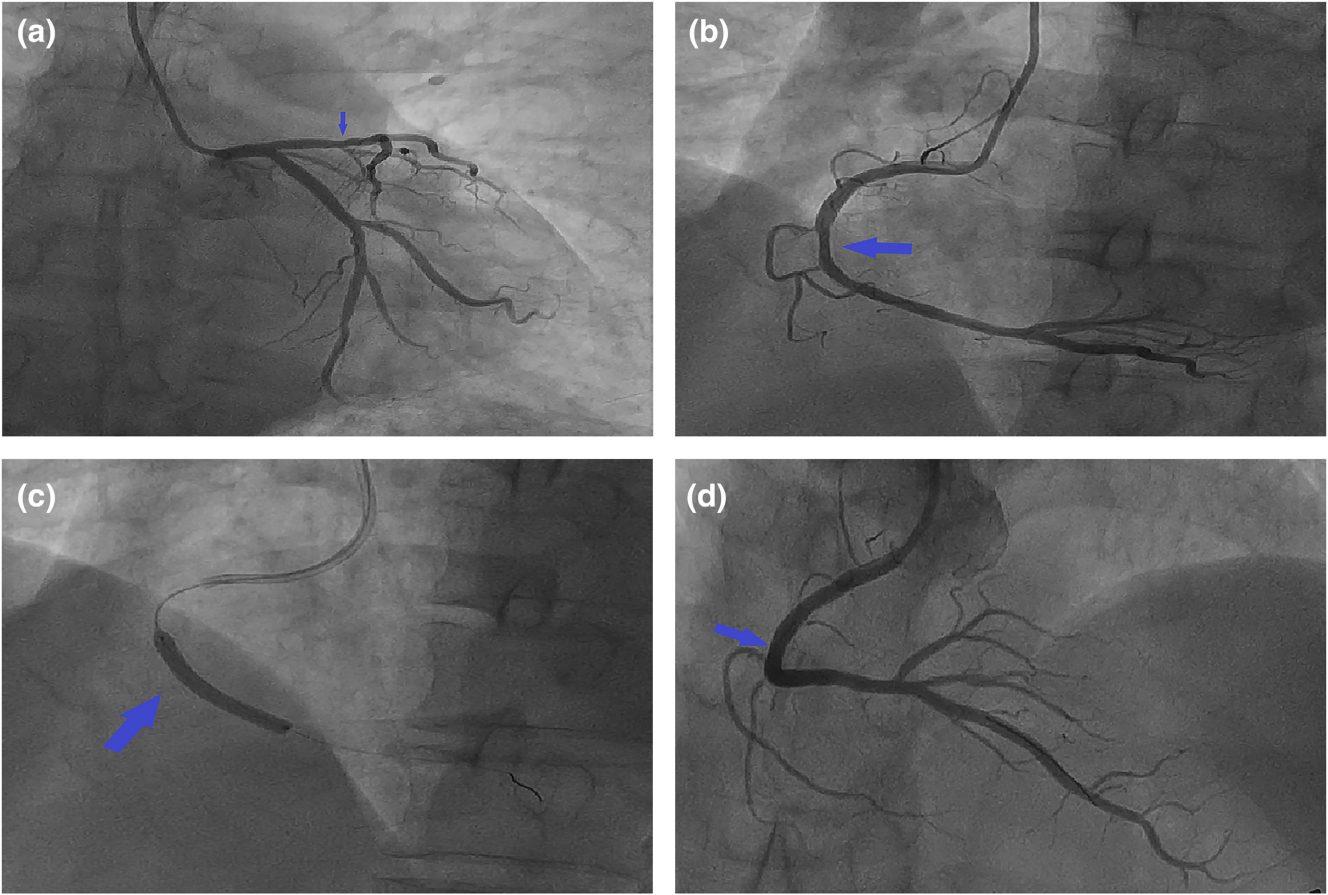
Coronary angiography. (a) The left main trunk was normal, the proximal segment of anterior descending branch was plaque, 30% localized stenosis in the middle segment (arrow), cyclotron branches scattered in plaques. (b) 70% of the RCA was narrowed in the middle and far segment (arrow). (c) A BRS (arrow) was implanted in the middle and distal segments of the RCA. (d) The blood of the RCA resumed to flow smoothly (arrow).

**FIGURE 3:**
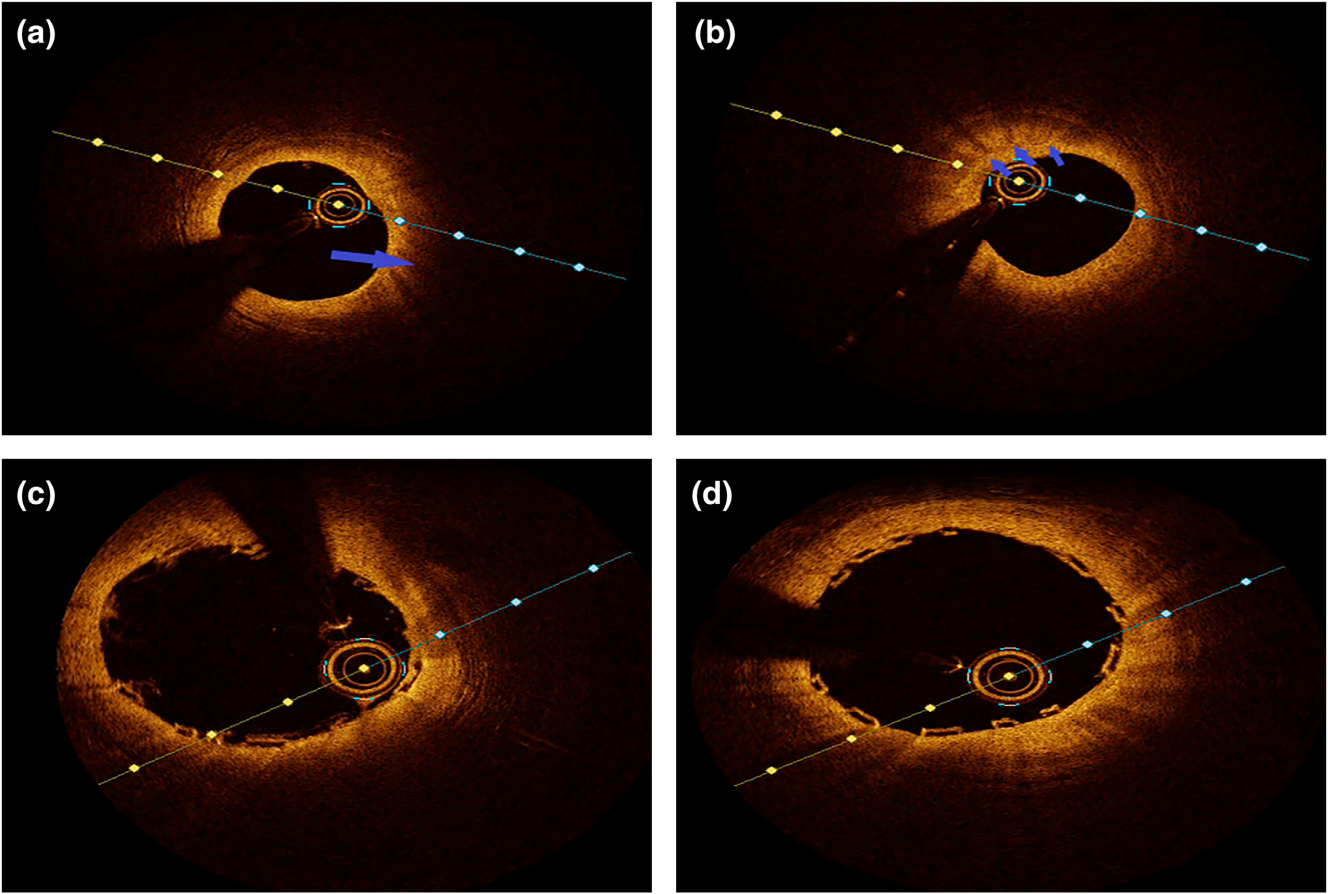
Optical coherence tomography. (a) Optical coherence tomography showed fibrous plaque at the middle and distal segment of the RCA (arrow). (b) The macrophage at the middle and distal segment of the RCA (arrow), and MLA 3.8 mm^2^ before the stent was implanted. (c) The stent was dilated and adhered well, with no interlayer at the upper end. (d) The stent was dilated and adhered well, with no interlayer at the lower end, and MSA 8.14 mm^2^.

**FIGURE 4:**
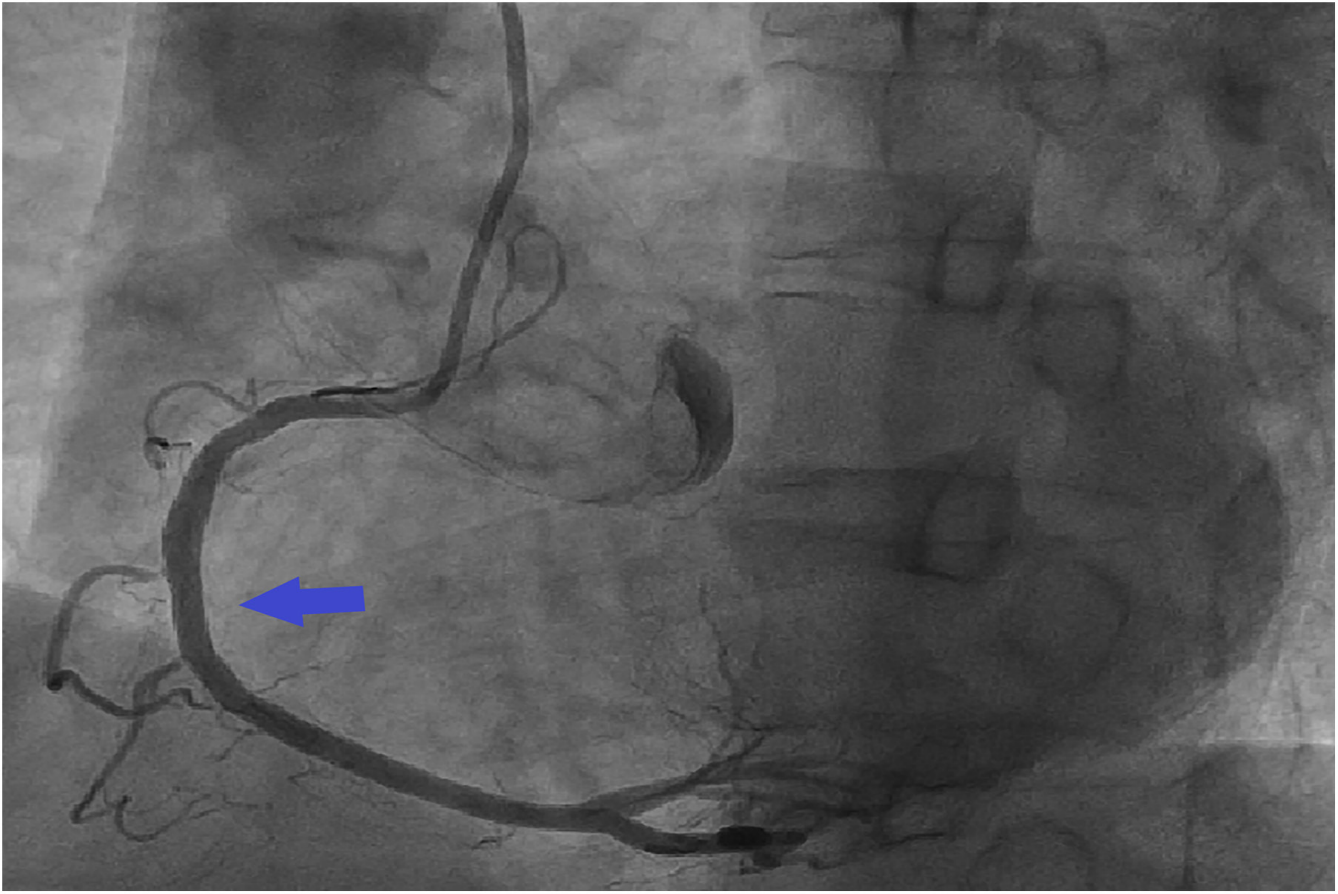
Repeated coronary angiography showed that the stent was good and the blood flow was normal (arrow).

Since the possibility of unstable angina pectoris caused by CAS could not be excluded, the patient was recommended to undergo CAG for evaluation. CAG was performed after obtaining the informed consent of the patient and his family. The results of CAG showed that the RCA was dominant, the left main trunk was normal, the proximal segment of anterior descending branch was plaque, 30% localized stenosis in the middle segment, cyclotron branches scattered in plaques (Figure 2a), and 70% of the RCA was narrowed in the middle and far segment (Figure 2b). OCT was performed after obtaining consent. After the guide wire was inserted into the distal end of the RCA, OCT showed fibrous plaque and macrophage at the middle and distal segment of the RCA, and MLA 3.8 mm^2^ (Figure 3a,b). Combining the patient's clinical manifestations and results of treadmill test, CAG and OCT, doctors considered that he had RCA stenosis combined with CAS. It was recommended that he be implanted with bioresorbable scaffold (BRS). A BRS was implanted in the middle and distal segments of the RCA (Figure 2c), OCT showed that the stent was dilated and adhered well, with no interlayer at the both ends, and MSA 8.14 mm^2^ (Figure 3c,d), the blood of the RCA resumed to flow smoothly (Figure 2d). After operation, the ECG showed sinus heart rate, and roughly normal ECG (Figure 1g); meanwhile, he was given isosorbide mononitrate sustained release tablets, diltiazem hydrochloride sustained release tablets, aspirin enteric‐coated tablets, ticagrelor tablets, atorvastatin calcium tablets, and sodium rabeprazole enteric‐coated tablets. He was discharged after recovering and instructed to take medication regularly. One year later, the patient did not complain of chest tightness and other discomfort. The repeated CAG showed that the stent without stenosis and the blood flow was normal (Figure 4).

## 2 DISCUSSION

CAS attack can not only directly lead to myocardial ischemia but also spasm itself can promote the rupture of vulnerable plaques, reduce coronary blood flow rate, activate the coagulation system and platelets, and other adverse outcomes (Kim et al., 2022). A variety of pathogenic mechanisms, such as vascular endothelial cell dysfunction, hyperresponsiveness of smooth muscle cells, abnormal production of growth factors and abnormal adventitia, magnesium deficiency and various genetic polymorphisms, have been confirmed to be involved in the occurrence of CAS (Matta et al., 2020; Picard et al., 2019). Intracoronary ultrasound imaging (IVUS) and OCT studies show that less plaque, more diffuse intimal thickening, no calcification, less lipid and necrotic core, cap‐like fibrous atheroma, and less cap fibrous atherosclerosis, thicker baseline medial width, smaller baseline lumen area, but more common baseline intimal bulge and negative remodeling at the site of spasm (Tsujita et al., 2013).

The treatment principle of CAS is to relieve the status of spasticity as soon as possible and actively treat the complications. With the popularization of coronary interventional diagnosis and treatment, reports of acute myocardial infarction (AMI) caused by CAS are increasing. Statistics show that about 10% of patients with AMI have no significant coronary artery stenosis, and CAS is considered to be one of the main causes of AMI (Park et al., 2018). The study by (Gaspardone et al., 1999) showed that symptoms were successfully controlled in six of nine patients with drug‐resistant CAS after coronary stenting at 6 months of follow‐up. In a single‐center analysis (Chu et al., 2013) including 21 patients with refractory variant angina who underwent coronary stenting, only 1 patient had persistent variant angina, 5 had occasional chest pain, and the remaining 15 had no symptom. Percutaneous coronary intervention in patients with documented coronary vasospasm is workable.

In order to avoid the permanent retention of metallic foreign bodies in the body, the concept of BRS has been proposed in recent years to be used in patients with stable coronary heart disease or low‐ and intermediate‐risk acute coronary syndrome (Zheng et al., 2022). BRS has the characteristics of wide trabeculae, which may inhibit the thrombus in the lesion from blocking the distal blood vessels. Secondly, BRS absorption may avoid delayed poor adhesion caused by extravascular thrombosis after metal stent implantation (Sabaté et al., 2016; Wu et al., 2020). In this CAS case, BRS was implanted under the guidance of treadmill test and OCT for treatment. The follow‐up results of the patient after 1 year showed that the BRS was good and the blood flow was normal. Therefore, it is hoped that more and more evidence‐based medical evidence supports the use of BRS in CAS patients.

## 3 CONCLUSION

In conclusion, clinicians should pay attention to the occurrence of CAS, which may be silent but fatal. Although there is currently no consensus on the ultimate treatment regimen for CAS, lifestyle changes such as smoking cessation and treatment with calcium channel blockers or nitrates remain the cornerstone of CAS treatment, in combination with other drugs such as statins or antiplatelet drugs may be more effective. Although BRS absorption might not prevent recurrent angina symptoms at the chronic phase, BRS is still an effective option for patients who do not respond to medical therapy and continue to experience recurrent angina attacks. Further large‐scale multicenter trials are needed in the future to explore final treatment options for patients with CAS.

## AUTHOR CONTRIBUTIONS

Yang Yang, Qianwei Huang, and Kai Zou: collection and collation of data. Xinghua Jiang and Jun Guo: analysis and interpretation of imaging. Yang Yang: writing of the first manuscript. Biming Zhan: study concept and critical revision of the manuscript for intellectual content. All authors contributed to this article and agreed to the final manuscript.

## CONFLICT OF INTEREST

The authors have no conflicts of interest to disclose.

## ETHICS STATEMENT

We identify that the ethics committee of the Second Affiliated Hospital of Nanchang University have approved the case, and that this case conforms to recognized standards, Declaration of Helsinki.

## Data Availability

Data sharing not applicable to this article as no datasets were generated or analysed during the current study.
